# Synthesis and characterization of single-phase epitaxial Cr_2_N thin films by reactive magnetron sputtering

**DOI:** 10.1007/s10853-018-2914-z

**Published:** 2018-09-18

**Authors:** M. A. Gharavi, G. Greczynski, F. Eriksson, J. Lu, B. Balke, D. Fournier, A. le Febvrier, C. Pallier, P. Eklund

**Affiliations:** 1 Thin Film Physics Division, Department of Physics, Chemistry and Biology (IFM), Linköping University, 581 83 Linköping, Sweden; 2 Universität Stuttgart, Institut für Materialwissenschaft - Chemische Materialsynthese, Heisenbergstr. 3, 70569 Stuttgart, Germany; 3 UMR 7588, Institut des NanoSciences de Paris, CNRS, Sorbonne Université, 4 Place Jussieu, 75005 Paris, France

## Abstract

Cr_2_N is commonly found as a minority phase or inclusion in stainless steel, CrN-based hard coatings, etc. However, studies on phase-pure material for characterization of fundamental properties are limited. Here, Cr_2_N thin films were deposited by reactive magnetron sputtering onto (0001) sapphire substrates. X-ray diffraction and pole figure texture analysis show Cr_2_N (0001) epitaxial growth. Scanning electron microscopy imaging shows a smooth surface, while transmission electron microscopy and X-ray reflectivity show a uniform and dense film with a density of 6.6 g cm^−3^, which is comparable to theoretical bulk values. Annealing the films in air at 400 °C for 96 h shows little signs of oxidation. Nano-indentation shows an elastic–plastic behavior with *H* = 18.9 GPa and *E*_r_ = 265 GPa. The moderate thermal conductivity is 12 W m^−1^ K^−1^, and the electrical resistivity is 70 μΩ cm. This combination of properties means that Cr_2_N may be of interest in applications such as protective coatings, diffusion barriers, capping layers and contact materials.

## Introduction

Chromium nitride (CrN) is a hard and corrosion resistant, semiconducting compound that has gained interest for various applications such as medical implants [1], silver luster decorative coatings [2], and wear-resistant coatings for cutting tools, especially when hot corrosion resistance is needed [3, 4]. It also has intriguing thermal and thermoelectric properties [5–8]. Chromium nitride is an interstitial compound, in which nitrogen atoms reside in the octahedral spaces between the chromium atoms in an fcc lattice (i.e., rock-salt cubic), making the compound susceptible to stoichiometry deficiency. This leads to a second interstitial compound: metallic dichromium nitride (Cr_2_N). Both phases have a silver luster when seen by the naked eye and can be used as decorative coatings. Unlike CrN however, Cr_2_N crystallizes in a hexagonal crystal structure and is usually reported as a secondary phase in either CrN hard coatings or steel [9–11]. Phase shifts between CrN and Cr_2_N have also been studied and are controlled by the partial pressure of nitrogen gas flow and deposition temperature [12]. In a previous paper [13] focused on monochromium nitride, we studied CrN thin films for its thermoelectric properties. These films were synthesized by reactive magnetron sputtering, and it was found that by decreasing the nitrogen content of the N_2_/Ar gas flow mixture, Cr_2_N nanoinclusions will form inside the CrN matrix. At a sufficiently low N_2_/Ar ratio, single-phase Cr_2_N could be synthesized. Thermoelectric measurements showed that Cr_2_N has a Seebeck coefficient of a few μV K^−1^. Also, the electrical resistivity slightly increases from 56 μΩ cm at room temperature up to 89μΩ cm  at 750 K, showing that Cr_2_N is metallic in nature.

Yan and Chen [14] used first principles calculations to predict a Young’s modulus of 356 GPa, suitable for hard-coating applications. This is due to the distance between N atom sites which are repulsive in nature, leading to enhanced stability of Cr_2_N. Wei et al. [15]. reported higher hardness properties for mixed phase CrN/Cr_2_N films compared to single-phase CrN. Single-phase Cr_2_N has also been studied [16, 17] for its mechanical properties. However, these films either are polycrystalline or have small amounts of Cr impurities. Additional research on the synthesis of polycrystalline CrN/Cr_2_N alloys [18] or CrN/Cr_2_N multilayers [19, 20] shows promising results for the wood cutting industry due to their suitable adhesion, wear resistance and hardness of approximately 22 GPa coupled with a relative low price in comparison with diamond or even titanium-based coatings.

In the present paper, we report on synthesis and property characterization of single-phase, epitaxial Cr_2_N. These films were deposited by reactive magnetron sputtering, did not show any phase impurities and are single crystal.

## Experimental details

The deposition process was carried out by reactive dc magnetron sputtering in a high vacuum chamber (base pressure: 3.0 × 10^−7^ mbar at deposition temperature). Details of the deposition setup can be found elsewhere [21]. The films were deposited onto (0001) sapphire substrates (10 × 10 × 0.5 mm^3^, ultrasonically cleaned in hellmanex (3 min), de-ionized water, acetone and ethanol (10 min each) and blown dry using N_2_ [22]. The substrates were heated 700 °C for 1 h prior to the deposition process and maintained until the end of the process. The films were deposited using a 7.5-cm Cr (Kurt J. Lesker, 99.95% pure) target and an argon/nitrogen gas mixture (99.9997% pure for both gases). The target power for chromium was kept constant and set at 60 W under a constant deposition pressure of 0.6 Pa (4.5 mTorr). The substrate holder was rotated with a speed of 17 rpm and was grounded. The deposition time was 120 min for the 17% nitrogen gas mixture (9 sccm N_2_, 45 sccm Ar).

X-ray diffraction (XRD) *θ*–2*θ* scans were carried out in a PANalytical X’Pert PRO diffractometer system equipped with a Cu K*α* source operated at 45 kV and 40 mA. The incident optics was a Bragg–Brentano module including a 0.5° divergence slit and a 0.5° anti-scatter slit, and the diffracted optics included a 5.0-mm anti-scatter slit and 0.04-rad soller slits. The PreFIX detector was set to one-dimensional scanning line mode. Step sizes and collection times per step were 0.004° and 2 s, respectively. High-resolution XRD and pole figure measurements were performed in a PANalytical EMPYREAN diffractometer operated at 45 kV and 40 mA. For HRXRD (line focus mode), the incident optics was a hybrid monochromator 2 bounce Ge (220) module including a 0.5° divergence slit, and the diffracted optics was a 3 bounce Ge (220) symmetrical analyzer. The XRD step sizes and collection times per step were 0.001° and 0.8 s, respectively. For the pole figure measurements (point focus mode), a 2 × 2 mm^2^ crossed slit X-ray lens and a parallel plate collimator as the incident and diffracted beam optics were used, respectively. Pole figures of the Cr_2_N (0002) and ($\bar{1}\bar{1}21$) peaks were acquired in the tilt-angle (*ψ*) range between 0 and 85° and azimuth-angle (*φ*) range between 0 and 360° with steps of 5° for both *ψ* and *φ* and a collection time of 1 s.

The chemical composition of the Cr_2_N films is deduced from X-ray photoelectron spectroscopy (XPS, Axis Ultra DLD, Kratos Analytical, UK) equipped with a monochromatic Al($K_{\alpha }$) X-ray radiation (*hν* = 1486.6 eV) source. The base pressure in the analysis chamber during acquisition was less than 1 × 10^−9^ mbar. Survey spectra as well as XPS core level spectra of the Cr 2p, N 1s and O 1s regions were recorded on as-deposited samples and after sputter cleaning for 600 s with a 0.5 keV Ar^+^ ion beam carried out to remove the surface oxygen layer that grows upon air exposure. The Ar^+^ beam had an incidence angle of 20° and was rastered over an area of 3 × 3 mm^2^. The core level spectra recorded after Ar^+^ etching were used to extract the chemical composition of the CrN coatings. Here, a Shirley-type background was subtracted, and elemental cross sections provided by Kratos Analytical were applied. Due to preferential sputtering of nitrogen, a 3% error bar is expected [23]. XPS sputter depth profiles in 0.8 nm steps were also performed on samples annealed in air to evaluate resistance toward oxidation. In this case, the Ar^+^ ion energy was 0.5 keV. Oxidation resistance testing was performed by heating a Cr_2_N thin film (epitaxial CrN and polycrystalline CrN thin films were included as reference samples) on a standard laboratory hot plate at 400 °C for 96 h under atmospheric conditions. The hot-plate temperature was measured with a thermocouple connected to a multimeter.

A JEOL 2200FS operating at 200 kV was used for transmission electron microscopy (TEM), high-resolution TEM (HRTEM), and selected area electron diffraction (SAED). Cross-sectional samples for the TEM were prepared by mechanically polishing them down to 55 μm by hand. Ion milling was then applied using a Gatan Precision Ion Polishing System (PIPS) with a 5 keV Ar^+^ ion beam angled at 8° of incidence relative to the sample surface for 2 h and 5° for 4 h. Once electron transparency was reached, a final ion milling step was applied with lower energy ion beams (2 keV) for 30 min.

Sample surface morphologies were obtained by a LEO 1550 Gemini scanning electron microscope (SEM). The acceleration voltage was set to 5 kV.

The electrical resistivity of all samples was obtained by measuring the sheet resistance of the films with a four-point-probe Jandel RM3000 station and multiplying the sheet resistance with the film thickness of approximately 450 nm (obtained from cross-sectional TEM imaging). Such measurements include a ± 10 nm error bar.

Temperature-dependent Hall carrier concentrations were determined on 5 × 5 mm^2^ substrates using the IPM-HT Hall-900 K system (developed and constructed by Fraunhofer IPM in Freiburg, Germany) and includes a 10% error bar. Thermal conductivity of selected samples was determined by modulated thermoreflectance microscopy (MTRM). In this setup, a pump beam at 532 nm (delivered by a Cobalt MLD laser and with the intensity modulated by an acousto-optical modulator at a frequency f) is focused on the surface of the sample with an objective lens (N. A. = 0.5). The samples were coated by a 100 nm gold layer, ensuring that the heat source is located at the surface. Thermal waves are excited in the sample and monitored by the reflectivity surface change recorded around the pump location by another focused laser beam. A 488 nm Oxxius laser is used to maximize the probe sensitivity toward the thermal field in the gold cap layer. A photodiode and a lock-in amplifier recorded the AC reflectivity component, in a frequency range between 1 kHz and 1 MHz. Finally, the experimental profiles of the amplitude and the phase of the reflected probe beam were fitted according to a standard Fourier diffusion law to extract the thermal conductivity of the films and include a measurement error of 15% [24–30].

The mechanical properties (hardness and elastic modulus) of the Cr_2_N films were investigated by nano-indentation using a Triboindenter TI 950 (Hysitron) and a Berkovich diamond tip with an apex radius of 100 nm. The mechanical response of the thin films was recorded for 30 nanoindents at a constrained load of 1300 μN in order to respect the rule of thumb of a maximal penetration depth below 10% of the film thickness. The data were analyzed using the approach of Oliver and Pharr [31].

## Results

**Figure 1:**
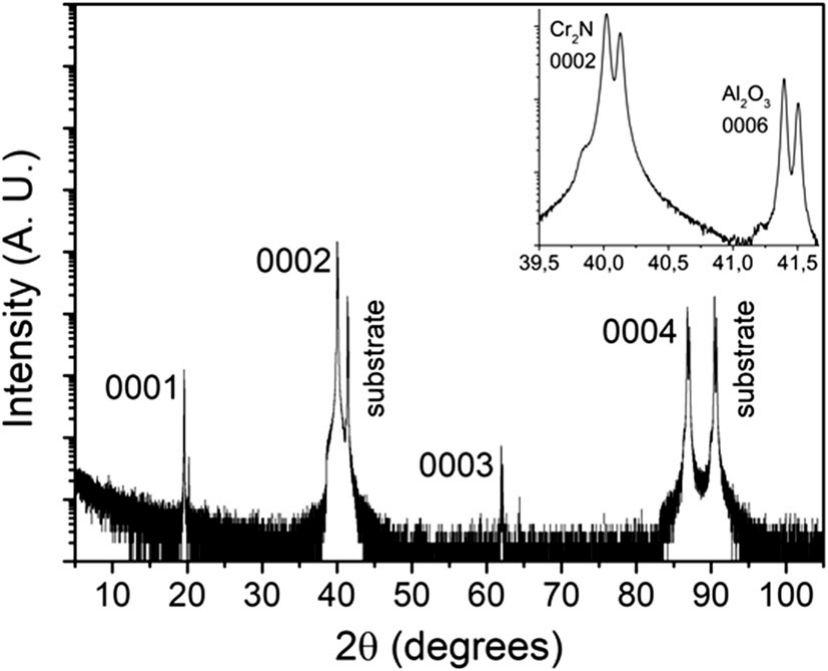
XRD results of Cr_2_N deposited on sapphire substrates. Inset: close-up image of the Cr_2_N 0002 and Al_2_O_3_ 0006 peaks. Note the $K_{\alpha 1}$–$K_{\alpha 2}$ peak splitting indicating high crystal quality

Figure 1 shows a *θ*–2*θ* scan of Cr_2_N film on a c-cut sapphire substrate. Only peaks of the {0001} family are observed from the film, showing *c*-axis oriented growth. The inset is a close-up image of the Cr_2_N $\left( {0002} \right)$ peak and the sapphire $\left( {0006} \right)$ peak and shows $K_{\alpha 1}$–$K_{\alpha 2}$ peak splitting.

**Figure 2:**
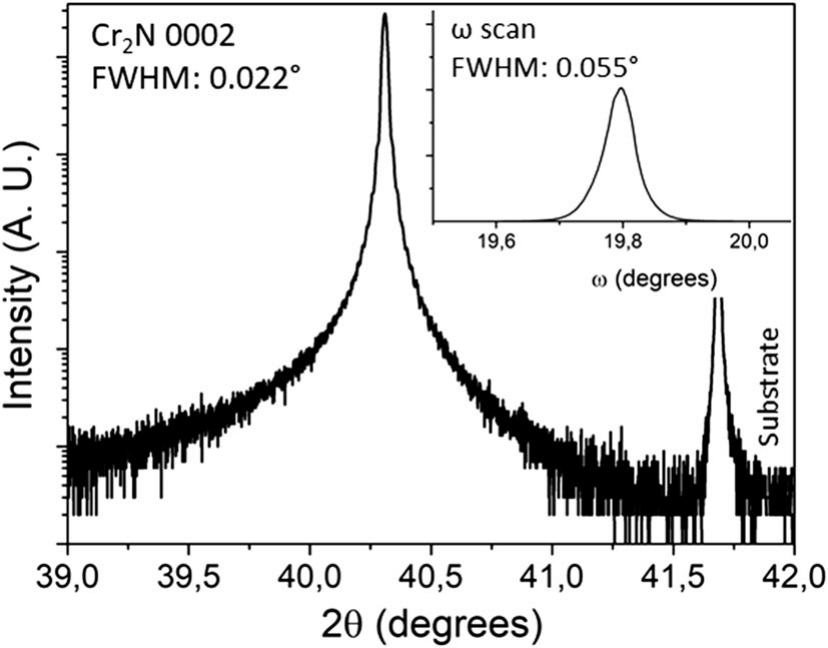
HRXRD measurement of Cr_2_N 0002 peak. Inset shows the rocking curve scan of Cr_2_N 0002

Figure 2 shows a high-resolution XRD scan of the Cr_2_N 0002 peak. The peak shows Laue oscillations (layer thickness fringes). The full width at half maximum (FWHM) is 0.022° compared to the FWHM value of 0.007° for the substrate peak. Rocking curve measurements of both the Cr_2_N 0002 (Fig. 2 inset) and 0004 peaks show FWHM values of 0.055° and 0.044°, respectively.

**Figure 3:**
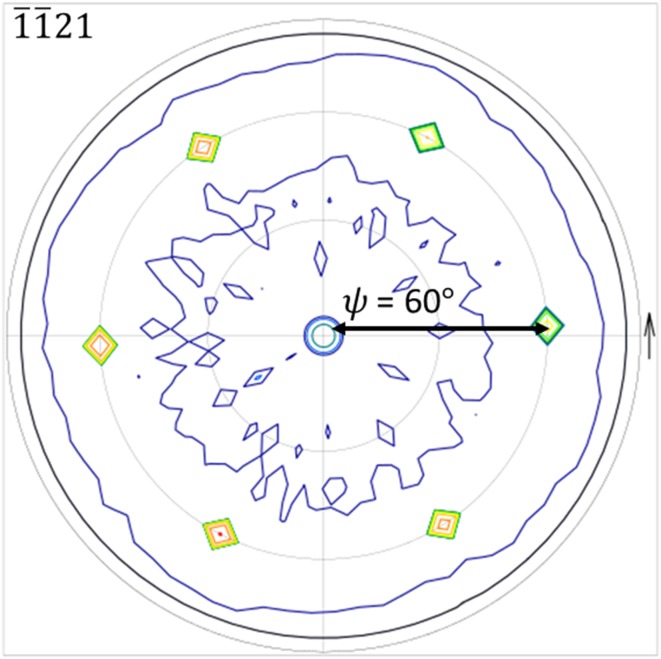
Pole figure texture analysis of Cr_2_N at $2\theta = 42.76^\circ$ corresponding to the $\bar{1}\bar{1}21$ peak

Figure 3 shows a pole figure measurement of the Cr_2_N thin film. The pole figure is performed at $2\theta = 42.76^\circ$ corresponding to the $\bar{1}\bar{1}21$ peak. In this case, six high-intensity poles at $\psi = 60^\circ$ surround a low-intensity pole in the center corresponding to the 0002 peak. Thus, a ($1\bar{2}10$)(0001)Cr_2_N//($1\bar{2}10$)($0001$)Al_2_O_3_ and [$1\bar{1}00$] Cr_2_N//[$1\bar{1}00$] Al_2_O_3_ epitaxial relationship leading to a single-crystal film on top of a single-crystal substrate is observed.

**Figure 4:**
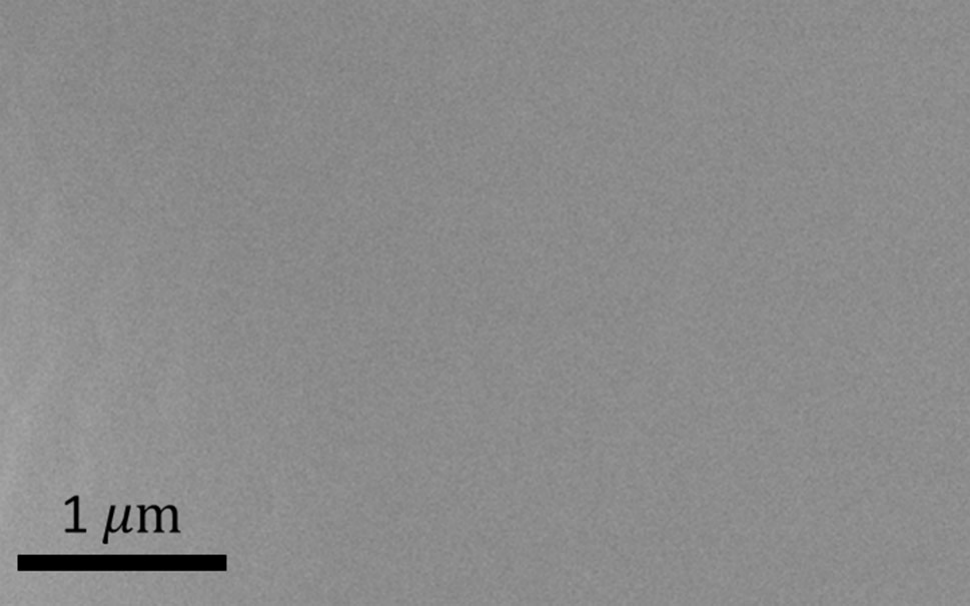
SEM image of Cr_2_N surface

XPS measurements show that the Cr_2_N film is stoichiometric (Cr 2p = 69%, N 1s = 30%) and contain approximately 1% oxygen and a negligible amount of carbon. Figure 4 shows an SEM image of the film surface. The film surface is smooth and featureless, consistent with the observed epitaxial growth and the high surface diffusion at 700 °C (the deposition temperature).

**Figure 5:**
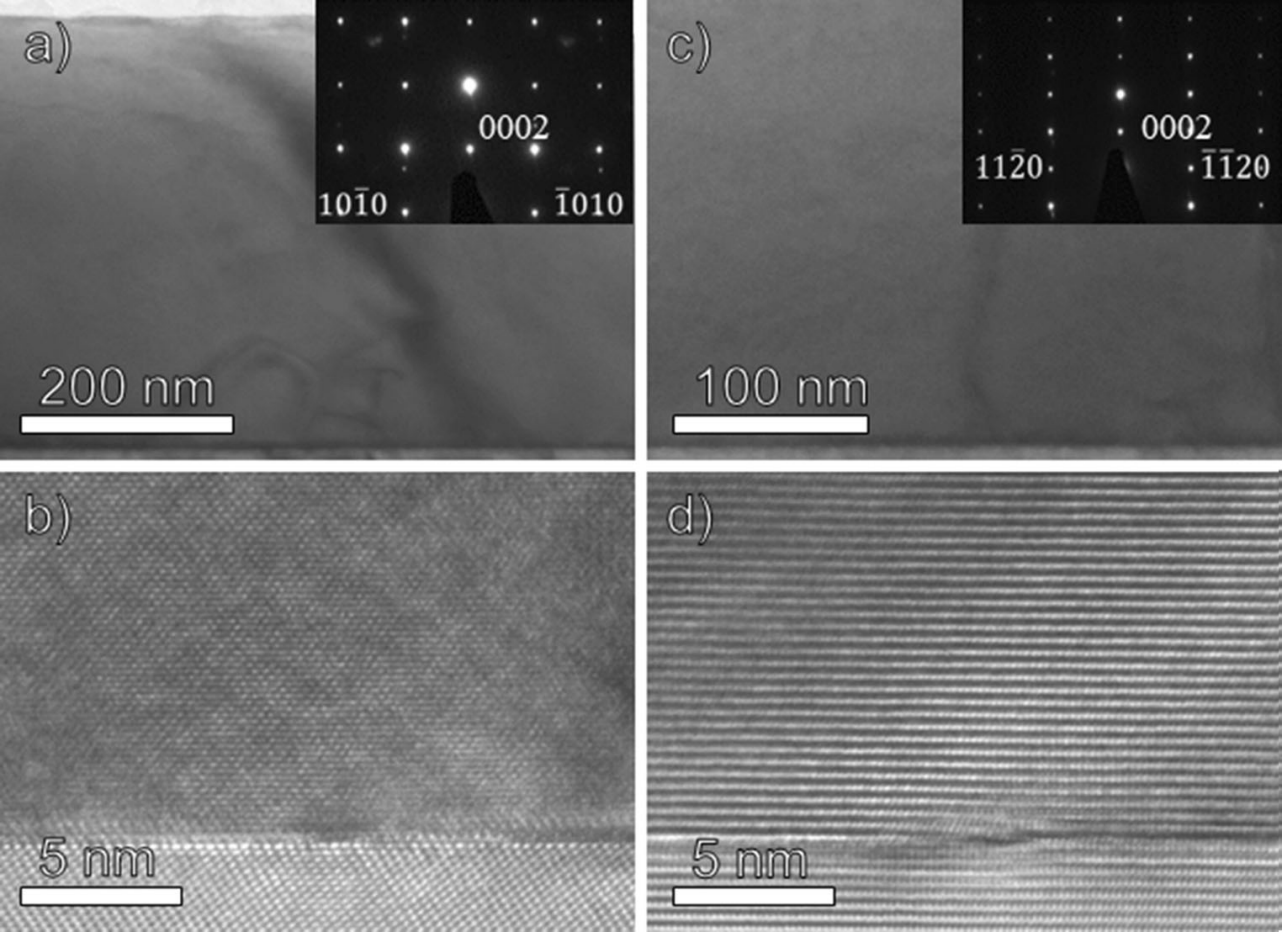
TEM, HRTEM and SAED images of Cr_2_N showing a dense and single-crystal film. **a** and **b** TEM and HRTEM image of the film in the $\left[ {1\bar{2}10} \right]$-zone axis including total film thickness (~ 450 nm) and epitaxial relationship with substrate. **c** and **d** TEM and HRTEM image of the film in the $\left[ {1\bar{1}00} \right]$-zone axis and epitaxial relationship with substrate. Note the layered structure of Cr_2_N when viewed from the $\left[ {1\bar{1}00} \right]$-zone axis

Figure 5 shows the TEM, HRTEM and SAED images for Cr_2_N. The images show a dense and single-crystal film with no grain boundaries in that scale. Epitaxy with the substrate is also seen which includes a sharp interface between film and substrate. In Fig. 5a, the TEM zone axis is in the [$1\bar{2}10$] direction, while in Fig. 5c, the TEM zone axis is in the [$1\bar{1}00$] direction.

Table 1 lists measured physical properties of the Cr_2_N film. According to the XRR measurements, the film has an arithmetic averaged roughness (*R*_a_) of 0.9 nm, which is comparable with polished, single-crystal substrates (0.5 nm). The same measurements also show a film density of 6.6 ${\text{g cm}}^{ - 3}$, which is comparable to the theoretical bulk value of 6.72 ${\text{g cm}}^{ - 3}$ [32].

**Table 1:** Collective data on the physical properties of Cr_2_N including film density, roughness, stoichiometry, hardness, elastic modulus, electrical resistivity, thermal conductivity and charge carrier density

Sample phase and thickness	Hardness and elastic modulus (GPa)	XRR measurements: roughness and density		Room temp. resistivity $\left( {{\mu \varOmega }\;{\text{cm}}} \right)$	Room temp. thermal conductivity $\left( {{\text{Wm}}^{ - 1} {\text{K}}^{ - 1} } \right)$	Charge carrier concentration $({\text{cm}}^{ - 3}$)	
		*R*_a_ (nm)	Density (g cm^−3^)			300 K	800 K
Hexagonal Cr_2_N ~ 450 nm	*H *= 18.9 ± 0.5 *E*_r_ = 265 ± 6	0.9 ± 0.1	6.6 ± 0.5	70 ± 7	12.0 ± 1.8	8.17 × 10^22^	28.70 × 10^22^

Room temperature in-plane electrical resistivity is measured to be 70 μΩ cm. This value is an average of four samples, with four measurements performed on each sample. The standard deviation in these measurements is 0.5 μΩ cm. Hall coefficient measurements show the concentration of charge carriers to be 8 × 10^22^ and 29 × 10^22^ ${\text{cm}}^{ - 3}$ at room temperature and 800 K, respectively. The room temperature in-plane thermal conductivity measurement is measured to be 12.0 W m^−1^ K^−1^, which is relatively low for metals and comparable to elemental mercury (8.4  W m^−1^ K^−1^) or nichrome alloys (between 10 and 20  W m^−1^ K^−1^ depending on alloy composition) [33, 34]. However, this value is three to four times larger than semiconducting CrN thin films [13].

**Figure 6:**
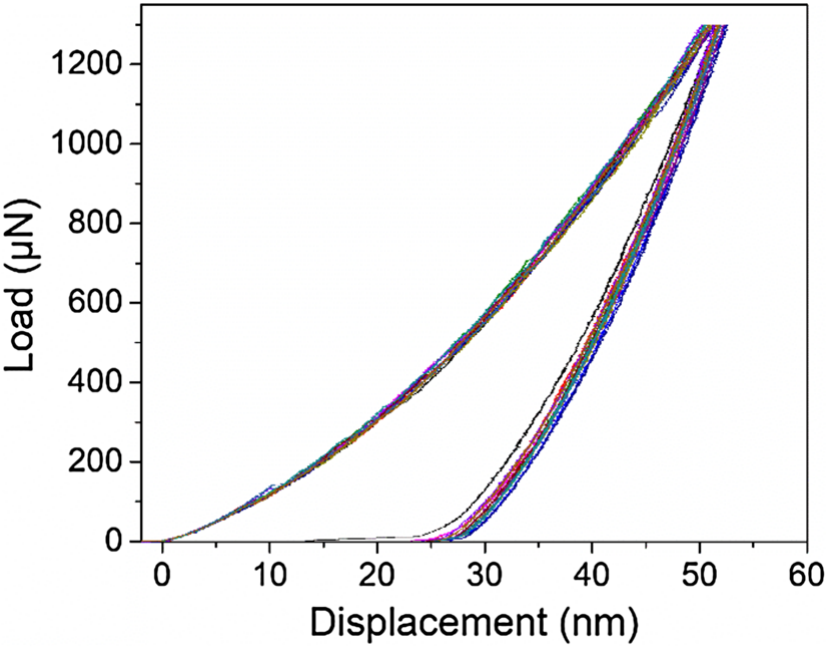
XPS results for the 400 °C oxidation test. Epitaxial CrN and epitaxial Cr_2_N were heated for 96 h, while as polycrystalline CrN was only heated for 48 h

Figure 6 shows the load versus displacement results for the nano-indentation measurement. Epitaxial Cr_2_N shows a semi elastic–plastic behavior due to the 25 nm displacement in the tip position before and after the load. The hardness and reduced elastic modulus of the film are $H = 18.9 \pm 0.5\;{\text{GPa}}$ and $E_{\text{r}} = 265 \pm 6\;{\text{GPa}}$, respectively. The error bar is based on the standard deviation in 28 measurements. In comparison, the hardness and reduced elastic modulus of titanium nitride (TiN) are $H = 20.0 \pm 0.8\;{\text{GPa}}$ and $E_{\text{r}} = 445 \pm 38  \;{\text{GPa}}$, respectively [35].

**Figure 7:**
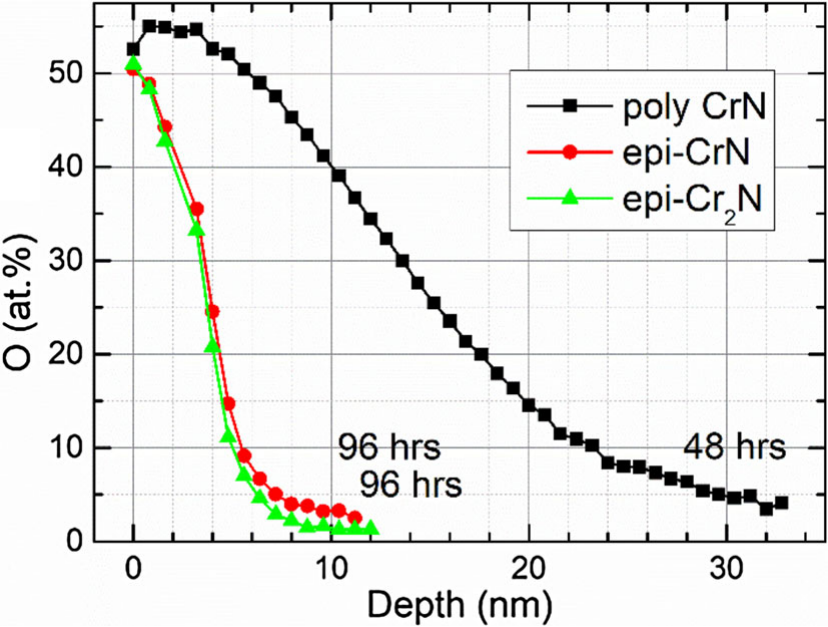
Load versus displacement plot for hardness measurements on Cr_2_N. The film shows an elastic–plastic behavior

Figure 7 shows the O concentration obtained from XPS sputter depth profiles performed in order to test the oxidation resistance during annealing in air for epitaxial Cr_2_N, epitaxial CrN and compare it to that of polycrystalline CrN used here as a reference sample. Epitaxial CrN [13] and epitaxial Cr_2_N were heated at 400 °C for 96 h, while the polycrystalline CrN test sample [36] was only heated for 48 h. XPS results reveal that in the case of epitaxial films the elevated oxygen concentration persists down to the depth of 8 nm from the surface, after which the signal saturates at the level of 1.3 at % which is due to residual O atoms implanted during the Ar^+^-etching process. In contrast, the polycrystalline CrN film exhibits higher O content even after removing first 30 nm from the surface and essentially never reaches the purity observed for the epitaxial films.

## Discussions

In general, all measurements indicate that Cr_2_N has been synthesized in the form of a dense, single-phase and single-crystal film. $K_{\alpha 1}$–$K_{\alpha 2}$ peak splitting in the low-resolution XRD measurements are visible in both film and substrate peaks indicating high crystal quality due to the minor energy difference between the $K_{\alpha 1}$–$K_{\alpha 2}$ X-rays, which would not be resolved for lower crystal quality. The HRXRD measurements show Laue oscillations (layer thickness fringes) which are only seen in very smooth films [37]. By using the equation $t = \frac{{\lambda \left( {n_{2} - n_{1} } \right)}}{{2\left( {\sin \theta_{2} - \sin \theta_{1} } \right)}}$ for two consecutive fringes, film thickness can be calculated where *t* is film thickness,$\lambda$ is the X-ray wavelength, $\theta$ is the diffraction angle of the fringe and *n* is the fringe number. In our case, film thickness is calculated to be approximately 470 nm. The rocking curve measurements on the (0002) and (0004) peaks have similar full width at half maximum values, indicating little to no mosaicity in the film growth. This is because misaligned crystallites would broaden the rocking curve measurement. Pole figure measurements also show discrete poles which contrasts pole figure rings seen in polycrystalline films [37]. Due to the very low lattice mismatch between Cr_2_N [38] and Al_2_O_3_ [39], an overlap between the a-axis and b-axis of Cr_2_N and Al_2_O_3_ leads to a featureless film as seen in the SEM image (Fig. 4). The TEM and HRTEM images (Fig. 5) show the film to be single crystal, and the XRR measurements show that the film density is close to theoretical bulk crystal values, which is expected for a single-crystal film.

Although the electrical resistivity and charge carrier concentrations of Cr_2_N are similar to a metal, thermal conductivity measurements show a relatively low value of 12.0 ${\text{W}}\;{\text{m}}^{ - 1} \;{\text{K}}^{ - 1}$ (Table 1). In comparison, the thermal conductivity of semiconducting CrN is approximately 4.0 ${\text{W}}\;{\text{m}}^{ - 1} \;{\text{K}}^{ - 1}$, while this value for insulating sapphire is approximately 30.0 ${\text{W}}\;{\text{m}}^{ - 1} \;{\text{K}}^{ - 1}$ [39]. According to the Wiedemann–Franz law, the product of the electrical resistivity and thermal conductivity is a constant: $\rho \kappa = LT$, where *L* is the Lorenz number (2.44 × 10^−8^ ${\text{W}}\Omega \;{\text{K}}^{ - 2}$) and *T* is temperature. For our case, *LT*/*ρ *= 10.5 ${\text{Wm}}^{ - 1} {\text{K}}^{ - 1}$, thus proving that the phonon contribution in Cr_2_N thermal conductivity is small compared to the electrical contribution (as is in other metals).

As for the oxidation test, the results show that after the annealing process, sputter cleaning of epitaxial CrN and epitaxial Cr_2_N will remove an 8 nm oxide layer compared to the 30 nm oxide layer of polycrystalline CrN (and which was only annealed for half the time), thus proving the advantage of single-crystal films compared to that of polycrystalline ones where oxidation may proceed through grain boundaries. These features (plus the silver luster of the film) could be suitable for decorative hard coatings, contact material [40, 41], capping and diffusion layer [42, 43] applications or used as tough oxidation resistant films [44, 45] similar to other transition metal nitrides.

## Conclusions

Single-phase, epitaxial dichromium nitride thin films were synthesized by reactive magnetron sputter deposition onto c-cut sapphire substrates. These films are single crystal, permitting the characterization of the fundamental properties of this material system. Cr_2_N is metallic with an electrical resistivity of 70 ${\mu \varOmega }\;{\text{cm}}$ at room temperature but a relatively low thermal conductivity value of 12.0 ${\text{W}}\,{\text{m}}^{ - 1} \,{\text{K}}^{ - 1}$. These values are comparable with elements such as mercury or alloys such as nichrome. Cr_2_N is shown to have an elastic–plastic behavior with a hardness and reduced elastic modulus of $H = 18.9 \pm 0.5\;{\text{GPa}}$ and $E{\text{r }} = 265 \pm 6\;{\text{GPa}}$, respectively, suitable for soft bearing surfaces. It is also oxidation resistant up to at least 400 °C for 96 h. This, plus its silver luster, suggests Cr_2_N thin films as an interesting material for decorative hard coatings, contact material, capping and diffusion layer applications.
